# DNA Repair Pathways in Cancer Therapy and Resistance

**DOI:** 10.3389/fphar.2020.629266

**Published:** 2021-02-08

**Authors:** Lan-ya Li, Yi-di Guan, Xi-sha Chen, Jin-ming Yang, Yan Cheng

**Affiliations:** ^1^ Department of Pharmacy, The Second Xiangya Hospital, Central South University, Changsha, China; ^2^ Xiangya School of Pharmaceutical Sciences, Central South University, Changsha, China; ^3^ Department of Cancer Biology and Toxicology, Department of Pharmacology, College of Medicine, Markey Cancer Center, University of Kentucky, Lexington, KY, United States

**Keywords:** DNA damage, DNA repair pathways, mitochondrial DNA, drug resistance, cancer therapy

## Abstract

DNA repair pathways are triggered to maintain genetic stability and integrity when mammalian cells are exposed to endogenous or exogenous DNA-damaging agents. The deregulation of DNA repair pathways is associated with the initiation and progression of cancer. As the primary anti-cancer therapies, ionizing radiation and chemotherapeutic agents induce cell death by directly or indirectly causing DNA damage, dysregulation of the DNA damage response may contribute to hypersensitivity or resistance of cancer cells to genotoxic agents and targeting DNA repair pathway can increase the tumor sensitivity to cancer therapies. Therefore, targeting DNA repair pathways may be a potential therapeutic approach for cancer treatment. A better understanding of the biology and the regulatory mechanisms of DNA repair pathways has the potential to facilitate the development of inhibitors of nuclear and mitochondria DNA repair pathways for enhancing anticancer effect of DNA damage-based therapy.

## The DNA Repair Pathways

**FIGURE 1:**
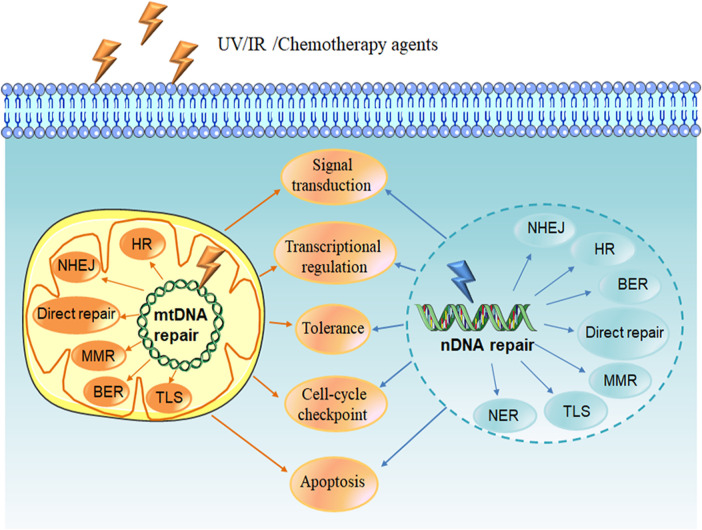
DNA damage response. DNA damage is caused by endogenous agent oxygen species (ROS) or exogenous agents such as UV light, ionizing radiation (IR) and chemotherapy agents. DNA damage response (DDR) is induced to deal with the lesions, including signal transduction, transcriptional regulation, cell-cycle checkpoints, induction of apoptosis, multiple DNA repair pathways as well as damage tolerance processes. DNA repair pathways include nuclear and mitochondrial DNA repair pathways. Direct repair, BER, MMR and recombinational repair (HR and NHEJ) are existence in both nuclear and mitochondrial repair systems. NER has been reported only appearance in nucleus, and the existence of TLS pathway in mitochondria is unknown. NDNA, nuclear DNA; MtDNA, mitochondrial DNA; BER, base excision repair; HR, homologous recombination repair; NHEJ, non-homologous end joining; MMR, mismatch repair; TLS, translesion synthesis; NER, nucleotide excision repair.

A variety of endogenous and exogenous DNA-damaging agents such as UV light, ionizing radiation (IR) and chemotherapeutic agents can lead to DNA lesions, including mismatches, single-strand breaks (SSBs), double-strand breaks (DSBs), chemical modifications of the bases or sugars, and interstrand or intrastrand cross-links. If the damage is not corrected, it will cause genomic instability and mutation, which is one of the cancer hallmarks (Hanahan and Weinberg, 2011). In order to prevent this situation, cells have evolved a series of mechanisms called DNA damage response (DDR) in order to deal with such lesions. DDR is a complex network that functions in different ways to target various DNA lesions, including signal transduction, transcriptional regulation, cell-cycle checkpoints, induction of apoptosis, damage tolerance processes, and multiple DNA repair pathways (Figure 1) (Giglia-Mari et al., 2011; Tian et al., 2015).

In mammalian cells, the two main organelles containing DNA are nucleus and mitochondria. Nuclear DNA (nDNA) repair systems are divided into the following major pathways: 1) direct reversal, which mainly repairs the lesion induced by alkylating agents, 2) base excision repair (BER), aiming at DNA breaks (SSBs) and non-bulky impaired DNA bases, 3) nucleotide excision repair (NER), correcting bulky, helix-distorting DNA lesions, 4) mismatch repair (MMR), repair of insertion/deletion loops (IDLs) and base-base mismatch, 5) recombinational repair, which is further divided into homologous recombination repair (HRR) and non-homologous end joining (NHEJ), primarily functioning at DNA double strand breaks, 6) alternative nonhomologous end joining (alt-NHEJ, MMEJ), involved in repair of DSBs, 7) translesion synthesis (TLS), which is more likely to be a DNA damage tolerance mechanism (Jackson and Bartek, 2009; Hosoya and Miyagawa, 2014). Mitochondrial DNA (mtDNA) repair pathways, including the direct reversal, BER, MMR, TLS and double-strand break repair (DSBR), can repair damaged DNA to maintain mitochondria genetic integrity, protect mtDNA against oxidative damage, and promote cell survival (Ohta, 2006; Saki and Prakash, 2017).

## Role of DNA Repair Pathways in Cancer Biology

DNA repair pathways play an important role in the maintenance of genome stability and integrity through correcting the impaired DNA that may contribute to carcinogenesis (Clementi et al., 2020). Numerous studies have indicated that certain cancers are associated with the defect or mutation in the proteins of nuclear or mitochondrial DNA repair pathways (Pearl et al., 2015; Cerrato et al., 2016). For example, the defect in the ATM–Chk2–p53 pathway, which plays a crucial role in DNA double-strand breaks repair, promoted glioblastoma multiforme (GBM) formation and contributed to GBMs radiation resistance (Squatrito et al., 2010). The human syndrome hereditary nonpolyposis colorectal cancer (HNPCC), which connects with high degrees of microsatellite instability, is caused by germline mutations in MMR genes, and the tumorigenesis of this disease is connected with the defect in the MMR pathway (Hampel et al., 2005). People who carry an MMR gene mutation have the increased risk of a wide variety of cancers than their noncarrier relatives (Win et al., 2012). Two important homologous recombination (HR) DNA repair-related genes, BRCA1 and BRCA2 germline mutant confer the genetic predisposition to breast, ovarian cancer and pancreatic cancer (Riaz et al., 2017). In addition, the tumor microenvironment characteristic of hypoxia, low pH and nutrient deficiency, can give rise to genomic instability and tumor progress through downregulating DNA repair pathway. It has been reported that hypoxic circumstance can result in the reduction of MLH1 expression, a core protein in the MMR pathway (Mihaylova et al., 2003). The downregulation of RAD51, a key mediator of HRR, was observed in multiple cancer cell types induced by hypoxia, suggesting that the hypoxic tumor microenvironment can suppress the HRR pathway to cause genetic instability (Bindra et al., 2004; Lu et al., 2011). Tumor hypoxia also regulated the DDR by driving alternative splicing (Memon et al., 2016). Study in human pulmonary epithelial cells has found that the acidic conditions delayed DNA damaging compounds benzo[a]pyrene (B[a]P) metabolism and inhibited NER capacity, ultimately enhanced B[a]P-induced DNA damage (Shi et al., 2017). Recent studies have shown that extracellular nutrients have significant effects on genome integrity. Glutamine is the main source of carbon and nitrogen for tumor cells. Lack of glutamine led to DNA alkylation damage by inhibiting ALKBH activity and increased the sensitivity of cancer cells to alkylating agents (Tran et al., 2017). Glucose starvation also enhanced radiosensitivity of tumor cells by reducing DNA double-strand break (DSB) repair (Ampferl et al., 2018). Thus, the dysregulation of DNA repair pathways can contribute to the development of cancer by promoting genomic instability and mutation in mammal cells.

## Targeting DNA Repair Pathways in Cancer Therapy

The most common cancer treatments, including chemo- or radiotherapy, are designed to induce cell death by direct or indirect DNA damage. However, tumor cells can initiate DNA repair pathways to resist these anticancer agents during chemo- or radiotherapy. Therefore, combination of the nuclear or mitochondrial DNA repair pathway inhibitors with anticancer agents may increase the tumor cell sensitivity to these agents.

### O-6-Methylguanine-DNA Methyltransferase (MGMT)

The role of MGMT is to remove alkyl adducts from the O^6^ position of guanine. Thus, the protective effect of MGMT could diminish the cytotoxic effects of alkylating agents (Middleton and Margison, 2003), suggesting that MGMT activity is likely to be a useful marker of the sensitivity of cancer cells to alkylating agents. It has been reported that high MGMT expression in tumor cell is associated with the resistance to 1,3- bis- (2-chloroethyl) -1- nitrosourea (BCNU) and temozolomide (TMZ) (Happold et al., 2018; Hsu et al., 2018), which target the O^6^-position of guanine, resulting in cytotoxic and mutagenic DNA adducts (Rabik et al., 2006). Recently, researchers found that MGMT-mediated the resistance to DNA alkylating agents in cancer cell is profoundly dependent on the DNA repair enzyme PARP. Combination of temozolomide with PARP inhibitors (PARPi) in MGMT-positive cancer cells enhanced the anticancer effects (Erice et al., 2015; Jue et al., 2017).

The inactivation of MGMT in tumor cells has been appreciated as a therapeutic target for sensitizing cells to O^6^-alkylating agents (Maki et al., 2005). *In vitro* and *in vivo* studies demonstrated that O^6^-Benzylguanine (O^6^-BG), a typical pseudo-substrate that was developed to inactivate MGMT, in combination with O^6^-alkylating agents increased the therapeutic efficacy of chemotherapeutic alkylating agents (Maki, Murakami, 2005). Lomeguatrib (called O^6^-(4-bromothenyl) guanine, as well as PaTrin-2), another pseudo-substrate tested in clinical trials, has been shown to increase the therapeutic index of methylating agent temozolomide in nude mice bearing A375M human melanoma xenografts and patients with advanced solid tumors (Middleton et al., 2002; Ranson et al., 2006). Bobustuc GC et al. demonstrated that inhibition of MGMT suppressed the expression of survivin and enhanced the cytotoxicity of gemcitabine in pancreatic cancer (Bobustuc et al., 2015). Another approach to MGMT inactivation is to silence the MGMT gene expression through its promoter methylation. Several studies in animal models have suggested that the therapy of MGMT gene silence was able to overcome TMZ resistance and increase tumor cell death (Viel et al., 2013). Clinical study indicated that patients with glioblastoma containing a methylated MGMT promoter obtained more benefits from TMZ than those who did not have a methylated MGMT promoter (Hegi et al., 2005). Lately, it has been confirmed that MGMT gene methylation can be a biomarker for temozolomide (TMZ) treatment and a potent prognostic factor in patients with GBM (Kim et al., 2012; Iaccarino et al., 2015; Zhao et al., 2016; Binabaj et al., 2018). However, according to the data from National *Cancer* database (NCDB) indicated that only 4.9% of GBM patients have MGMT promoter methylation. Even though MGMT promoter methylation status has prognostic value, it is ignored in the United States (Lee et al., 2018). More researches need to conduct to identify the prognostic value of MGMT promoter methylation in tumor patients responding to alkylating agents.

### Base Excision Repair

A number of investigations have shown that inhibition of BER pathway can enhance the sensitivity of cancer cells to alkylating agents and radiotherapy (Neijenhuis et al., 2005; Gao et al., 2019). The primary methods to prevent the activity of BER pathway focus on the development of AP endonuclease 1 (APE1) or Poly (ADP-ribose) polymerase (PARP) inhibitors.

Several studies indicated that methoxyamine (MX), a small alkoxyamine that can bind with the free aldehyde of AP site to prevent APE1 cleavage at AP sites, thereby inhibiting APE-1 endonuclease activity. Combined treatment with chemotherapeutic alkylating agent such as TMZ and BCNU could reinforce the cytotoxicity of alkylating agent by targeting BER pathway (Liu et al., 2003; Montaldi and Sakamoto-Hojo, 2013). Recently, based on preclinical studies, several clinical trials were conducted, for example combination therapy with MX and TMZ in patients with advanced solid tumors has completed (NCT00892385). Currently, phase Ⅰ clinical trials of MX in combination of TMZ is undergoing in patients with relapsed solid tumors and lymphomas (NCT01851369). MX combination with pemetrexed disodium, cisplatin, is now investigating in phase Ⅰ/II stage in patients with advanced malignant solid neoplasm (NCT02535312). Lucanthone, a topoisomerase II inhibitor as well as an APE1 endonuclease inhibitor, has been shown to reinforce the cell killing effect of alkylating agents in human breast cancer cell line MDA-MB-231 (Luo and Kelley, 2004). Lucanthone combination with radiation and TMZ in GBM patients was tested in phase Ⅱ clinical trial (NCT01587144). However, it was terminated in 2016. Another phase II clinical trial investigating lucanthone combination with radiation in patients with brain metastases from non-small cell lung cancer was withdrawn due to drug issues (NCT02014545).

**FIGURE 2:**
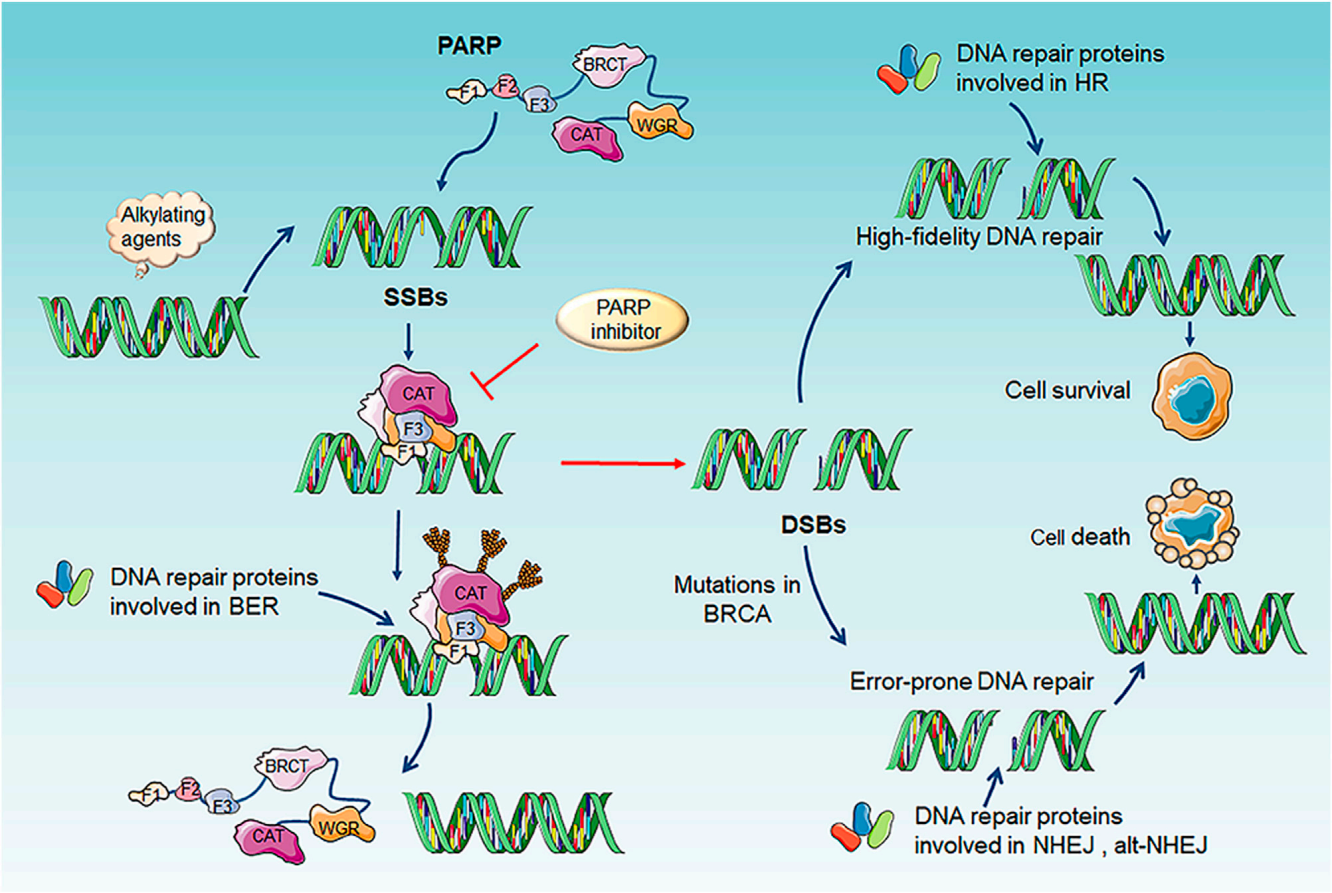
Mechanism and function of PARP and PARP inhibitors. The catalytic function of PARP1 is activated through binding to the SSBs site cuased by alkylating agents. Activated PARP1 undergo PARylation and recruitment of a serials of key DNA repair effectors involved in BER to repair DNA lesion. Finally, PARP1 release from DNA and regain inactive state. PARP inhibitors binds the catalytic site of PARP and impaired of the enzymatic activity of PARP which “trap” PARP1 on DNA, results in suppression of the catalytic cycle of PARP1 and BER. Trapping PARP1 on DNA lesion also collapses DNA replication fork, therefore transforming SSBs into genotoxic DSBs. This type of DNA lesion would normally induce HR for repairing damaged DNA. However, if HR-defective exist in tumor cells, including BRCA1/2 deficiency or mutation, another less effective and error-prone DSBs repair pathway NHEJ or alt-NHEJ could be utilized, which causing genomic instability, chromosomal fusions/translocations and subsequently inducing cell death. SSBs, single-strand breaks; DSB, double-strand break; BER, base excision repair; alt-NHEJ, alternative nonhomologous end joining; NHEJ, non-homologous end joining; HR, homologous recombination repair.

PARP family is composed of 17 members, of which PARP1 and PARP2 are well-recognized DNA damage sensors, especially PARP1. PARP1 detect the region of damaged DNA and play a key role in several DNA repair pathway including BER, HHR and MMEJ (Konecny and Kristeleit, 2016). While PARP1 is best studied in BER and the mechanism of PARP inhibitor (PARPi) is based on trapping PARP1 on SSBs DNA site to inhibit BER repair. Finally, it converted SSBs into DSBs and impelled cell death in HR-deficiency tumor, for example BRCA1/2 mutations, RAD51 deficiency (Figure 2) (Konecny and Kristeleit, 2016; Brown et al., 2017; Lord and Ashworth, 2017; Oplustil O’Connor et al., 2016). In 2005, two pre-clinical researches published in nature indicated that BRCA1 or BRCA2 deficient cells highly sensitized to PARP inhibition (Farmer et al., 2005; Bryant et al., 2005). Based on the concept of “synthetic lethality”-targeting either gene alone in a synthetic lethal pair is tolerated, but simultaneous targeting both genes is lethal, researchers applied PARPi to BRCA mutation tumors (Dhillon et al., 2016). Several clinical trials using PARPi including Olaparib, Veliparib, Rucaparib (Table 1) as monotherapy for the treatment of patients with germline BRCA1/2 mutation tumors including advanced breast cancer, ovarian cancer, pancreatic cancer and prostate cancer presented significantly antitumor effect (Kaufman et al., 2015; Robson et al., 2017; Moore et al., 2018; Golan et al., 2019). Olaparib as maintenance therapy also significantly prolonged progression-free survival in advanced ovarian cancer patients with HRD-positive tumors who have achieved first-line standard therapy including bevacizumab. It has been approved by FDA for utilization of Olaparib in patients with advanced germline BRCA-mutated ovarian cancer following three or more prior lines of chemotherapy (Kim et al., 2015). On May 19, 2020, the FDA also approved Olaparib for patients with metastatic castration-resistant prostate cancer (mCRPC) carrying HRR gene-mutated based on NCT02987543. PAPR1 inhibitors in combination with IR or with other different anticancer agents are currently undergoing clinical trials for treatment of patients with BRCA1/2 mutation or HRR-deficiency advanced solid tumors, which shown promising clinical activity (Bang et al., 2017; Wilson et al., 2017; Loibl et al., 2018; Coleman et al., 2019; Farago et al., 2019; Konstantinopoulos et al., 2019; Liu et al., 2019).

**TABLE 1:** DNA repair pathway inhibitors in current clinical trials.

Targeting protein	DNA repair pathway	Inhibitors	Clinical status	Disease state	Intervention/treatment	NCT number	Status
**PARP1/PARP2**	**BER**	**Olaparib (AZD-2281)**	**Phase II**	**Metastatic renal cell carcinoma with DNA repair gene mutations**	**Olaparib**	**NCT03786796**	**Recruiting**
			**Phase II**	**Mesothelioma with homologous recombination deficiency**	**Olaparib**	**NCT04515836**	**Not yet recruiting**
			**Phase II**	**Non-BRCA metastatic breast cancer (MBC)**	**Olaparib**	**NCT03367689**	**Recruiting**
			**Phase II**	**Metastatic urothelial cancer with somatic DNA damage response (DDR) alterations**	**Olaparib**	**NCT03448718**	**Recruiting**
			**Phase II**	**Metastatic breast cancer with BRCA1 and/or 2 promoter methylation**	**Olaparib**	**NCT03205761**	**Recruiting**
			**Phase II**	**Cisplatin-resistant germ cell tumor**	**Olaparib**	**NCT02533765**	**Active, not recruiting**
			**Phase I**	**Advanced cancer**	**Olaparib, AZD5363**	**NCT02338622**	**Completed**
			**Phase I**	**Triple negative breast cancer (TNBC)**	**Olaparib, radiation therapy**	**NCT03109080**	**Active, not recruiting**
			**Phase Ⅲ**	**HER2-ve metastatic breast cancer patient**	**Olaparib**	**NCT03286842**	**Active, not recruiting**
			**Phase Ⅳ**	**BRCA or HRR + mutated ovarian cancer**	**Olaparib**	**NCT02476968**	**Active, not recruiting**
		**Niraparib**	**Phase I**	**Advanced solid tumors**	**Niraparib**	**NCT03497429**	**Completed**
			**Phase II**	**Uterine serous carcinoma**	**Niraparib**	**NCT04080284**	**Recruiting**
			**Phase I**	**EGFR-mutated advanced lung cancer**	**Niraparib, osimertinib**	**NCT03891615**	**Recruiting**
			**Phase II**	**Pancreatic cancer**	**Niraparib**	**NCT03601923**	**Recruiting**
			**Phase I**	**Solid tumors**	**SYD985, niraparib**	**NCT04235101**	**Recruiting**
			**Phase Ⅲ**	**Ovarian cancer**	**Nirapairb**	**NCT03709316**	**Recruiting**
		**Talazoparib**	**Phase I**	**BRCA mutation-associated breast and ovarian cancers, pancreatic and small cell lung cancer**	**Talazoparib**	**NCT01286987**	**Completed**
			**Phase I**	**Leukemia with cohesin complex mutation**	**Talazoparib**	**NCT03974217**	**Recruiting**
			**Phase II**	**Advanced cancer with DNA repair variations**	**Talazoparib**	**NCT04550494**	**Not yet recruiting**
			**Phase II**	**Triple negative breast cancer**	**Talazoparib, ZEN003694**	**NCT03901469**	**Recruiting**
		**Veliparib (ABT-888)**	**Phase I**	**Pancreatic cancer**	**Veliparib, gemcitabine, radiation**	**NCT01908478**	**Completed**
			**Phase I**	**Refractory Solid Tumors**	**Veliparib, VX-970, cisplatin**	**NCT02723864**	**Active, not recruiting**
			**Phase II**	**Malignant glioma without H3 K27M or BRAFV600 mutations**	**Radiation, temozolomide, veliparib**	**NCT03581292**	**Recruiting**
			**Phase II**	**Metastatic breast cancer with BRCA1/2 gene mutation**	**ABT-888, temozolomide**	**NCT01009788**	**Active, not recruiting**
			**Phase II**	**Refractory testicular germ cell cancer**	**Gemcitabine, carboplatin, veliparib**	**NCT02860819**	**Active, not recruiting**
			**Phase I**	**Advanced malignant solid neoplasm**	**Dinaciclib, veliparib**	**NCT01434316**	**Recruiting**
		**Rucaparib**	**Phase I**	**Advanced solid tumor**	**Rucaparib, camsylate**	**NCT03521037**	**Active, not recruiting**
			**Phase II**	**Nonmetastatic, hormone-sensitive prostate cancer**	**Rucaparib**	**NCT03533946**	**Recruiting**
			**Phase I**	**Metastatic castration resistant prostate cancer**	**Rucaparib, enzalutamide, abiraterone**	**NCT04179396**	**Recruiting**
			**Phase II**	**High-grade serous or endometroid ovarian cancer**	**Rucaparib, nivolumab**	**NCT03824704**	**Active, not recruiting**
			**Phase II**	**Solid tumors and with deleterious mutations in HRR genes**	**Rucaparib**	**NCT04171700**	**Recruiting**
		**2X-121**	**Phase II**	**Metastatic breast cancer**	**2X-121**	**NCT03562832**	**Active, not recruiting**
**APE1**	**BER**	**Methoxyamine (TRC102)**	**Phase I/II**	**Relapsed solid tumors and lymphomas**	**TRC102**	**NCT01851369**	**Recruiting**
			**Phase I/II**	**Solid tumors or mesothelioma**	**Cisplatin, methoxyamine, pemetrexed disodium**	**NCT02535312**
			**Phase I**	**Stage IIIA-IV non-small cell lung cancer**	**Radiation, cisplatin**	**NCT02535325**	**Active, not recruiting**
**APE1/Ref-1**	**BER**	**APX3330 (E3330)**	**Phase I**	**Advanced solid tumors**	**APX3330**	**NCT03375086**	**Completed**
**DNA-PK**	**NHEJ**	**MSC2490484A (M3814)**	**Phase I**	**Locally advanced rectal cancer**	**M3814, avelumab, radiation**	**NCT03724890**	**Recruiting**
			**Phase I**	**Advanced solid tumor**	**Radiation, cisplatin, MSC2490484A**	**NCT02516813**	**Recruiting**
			**Phase I/II**	**Locally advanced rectal cancer**	**M3814, capecitabine, radiation**	**NCT03770689**	**Recruiting**
		**VX-984 (M9831)**	**Phase I**	**Advanced solid tumor**	**IV pegylated liposomal doxorubicin, VX-984**	**NCT02644278**	**Completed**
**DNA-PK/mTOR**	**NHEJ**	**CC-115**	**Phase I**	**Advanced solid tumors, hematologic malignancies**	**CC-115**	**NCT01353625**	**Active, not recruiting**
**ATM**	**HR**	**AZD0156**	**Phase I**	**Advanced solid tumors**	**AZD0156, olaparib, irinotecan, fluorouracil, folinic acid**	**NCT02588105**	**Active, not recruiting**
		**AZD1390**	**Phase I**	**Brain cancer**	**Radiation, AZD1390**	**NCT03423628**	**Recruiting**
			**Phase I**	**Non small cell lung cancer**	**Radiation, olaparib, AZD1390**	**NCT04550104**	**Not yet recruiting**
**ATR**	**HR**	**AZD6738 (Ceralasertib)**	**Phase II**	**Biliary tract cancer**	**AZD6738, durvalumab**	**NCT04298008**	**Recruiting**
			**Phase I**	**Leukemia, myelodysplastic syndrome**	**AZD6738**	**NCT03770429**	**Recruiting**
			**Phase II**	**Relapsed small cell lung cancer subjects**	**Durvalumab, AZD6738**	**NCT04361825**	**Enrolling by invitation**
			**Phase II**	**Clear cell renal cell carcinoma, locally advanced pancreatic cancer, locally advanced malignant solid neoplasm**	**AZD6738, olaparib**	**NCT03682289**	**Recruiting**
			**Phase I**	**Refractory cancer**	**AZD6738, paclitaxel**	**NCT02630199**	**Recruiting**
			**Phase II**	**Recurrent ovarian cancer**	**Olaparib pill, AZD6738**	**NCT03462342**	**Recruiting**
			**Phase II**	**IDH1 and IDH2 mutant tumors**	**Ceralasertib, olaparib**	**NCT03878095**	**Recruiting**
		**VE-822 (VX-970, M6620, berzosertib)**	**Phase II**	**Solid tumor, leiomyosarcoma, osteosarcoma**	**M6620**	**NCT03718091**	**Recruiting**
			**Phase1/II**	**Small cell cancers and extrapulmonary small cell cancers**	**Topotecan, VX-970**	**NCT02487095**	**Recruiting**
			**Phase I**	**Refractory solid tumors**	**Veliparib, VX-970, cisplatin**	**NCT02723864**	**Active, not recruiting**
			**Phase II**	**Small cell lung cancers and small cell cancers outside of the lungs**	**Berzosertib, topotecan hydrochloride**	**NCT03896503**	**Recruiting**
			**Phase II**	**Metastatic urothelial cancer**	**Berzosertib, cisplatin, gemcitabine hydrochloride**	**NCT02567409**	**Active, not recruiting**
**CHK1**	**HR**	**Prexasertib**	**Phase II**	**Triple negative breast cancer**	**LY3023414, prexasertib**	**NCT04032080**	**Recruiting**
			**Phase II**	**Recurrent or refractory solid tumors**	**Prexasertib**	**NCT02808650**	**Active, not recruiting**
			**Phase I/II**	**Desmoplastic small round cell tumor, rhabdomyosarcoma**	**Prexasertib, irinotecan**	**NCT04095221**	**Recruiting**
			**Phase II**	**Platinum-resistant or refractory recurrent ovarian cancer**	**Prexasertib**	**NCT03414047**	**Active, not recruiting**
			**Phase I**	**Advanced solid tumors**	**Prexasertib, olaparib**	**NCT03057145**	**Active, not recruiting**
		**MK-8776**	**Phase I**	**Acute leukemias**	**MK-8776, cytarabine**	**NCT00907517**	**Terminated**
		**SRA737**	**Phase I/II**	**Advanced solid tumors**	**SRA737, gemcitabine, cisplatin**	**NCT02797977**	**Completed**
**WEE1**	**HR**	**Adavosertib (AZD1775)**	**Phase II**	**Uterine serous carcinoma**	**Adavosertib**	**NCT04590248**	**Not yet recruiting**
			**Phase I**	**Advanced solid tumors**	**Adavosertib**	**NCT04462952**	**Recruiting**
			**Phase I/II**	**Relapsed or refractory solid tumors**	**Adavosertib, irinotecan hydrochloride**	**NCT02095132**	**Active, not recruiting**
			**Phase I**	**Newly diagnosed or recurrent glioblastoma**	**Adavosertib, radiation therapy, temozolomide**	**NCT01849146**	**Active, not recruiting**

Abbreviations: PARP, Poly (ADP-ribose) polymerase; APE1, AP endonuclease 1; Ref-1, redox factor-1; DNA-PK, DNA-dependent protein kinase; mTOR, mammalian target of rapamycin; ATM, ataxia telangiectasia mutated; ATR, ataxia telangiectasia and Rad3-related; CHK1, checkpoint kinase 1; WEE1, Wee1-like protein kinase.

### Double Strand Breaks Repair

Among various DNA lesions, DSBs is the leading lethal damage that leads to cell death and genetic mutations. DNA-dependent protein kinase (DNA-PK), a member of the PI3K-related protein kinase (PIKK) family, is involved in DSBs repair pathway via non-homologous end joining (NHEJ) (Jette and Lees-Miller, 2015). It has been reported that DNA-PK activity plays a role in chemo-radiotherapy resistance (Wang Y. et al., 2018; Stefanski et al., 2019; Alikarami et al., 2017; Liu et al., 2015). Selective DNA-PK inhibitor have been developed, including NU7026 (Dolman et al., 2015), NU7441 (Yang et al., 2016), IC87361 and SU11752 (Shinohara et al., 2005). They could inhibit DSBs repair pathway and enhance the sensitivity of cancer cells to ionizing radiation or/and chemo-potentiation such as doxorubicin (Ciszewski et al., 2014). The combination of DNA-PK inhibitor M3814 with type II topoisomerase inhibitors, including doxorubicin, etoposide and pegylated liposomal doxorubicin, enhanced the efficacy of type II topoisomerase inhibitors in ovarian cancer xenografts (Wise et al., 2019). Several novel DNA-PK inhibitors including MSC2490484A, VX-984 (M9831), M3814 are under clinical trial as single-agent or combination with Chemo-radiotherapy (Table 2). Alexander K. Tsai *et al.* recently found that NU7441 combination with a multikinase inhibitor regorafenib altered immune microenvironment of melanomas and enhanced the efficacy of various immunotherapies (Tsai et al., 2017).

**TABLE 2:** Inhibitors of DNA repair pathway recently under preclinical studies.

Inhibitor	DNA repair pathway	Target	Application	References
**Lomeguatrib (PaTrin-2)**	**Direct repair**	**MGMT**	**Pancreatic cancer cells; combination with HDACis in ovarian cancer**	**Wu et al. (2019)**, **Shi et al. (2020)**
**Lucanthone**	**BER**	**APE1**	**Glioblastoma multiforme (GBM) cell**	**Chowdhury et al. (2015)**
**CRT0044876**	**BER**	**APE1**	**Colon cancer cell lines**	**Seo and Kinsella (2009)**
**Methoxyamine**	**BER**	**APE1**	**Combination with pemetrexed in non-small-cell lung cancer cells and xenografts**	**Oleinick et al. (2016)**
**APX3330 (E3330)**	**BER**	**APE1/Ref-1**	**Bladder cancer**	**(Fishel et al., 2019)**
**RI-1**	**HR**	**RAD51**	**Combination with olaparib in breast cancer cells with wild-type PTEN; combination with radiation in glioma stem cells**	**King et al. (2017)**, **Zhao et al. (2017)**
**B02**	**HR**	**RAD51**	**Combination with radiation in glioma stem cells; combination with clinically approved anticancer agents in breast cancer cell**	**Huang and Mazin (2014)**, **King et al. (2017)**
**AG-14361**	**BER**	**PARP1**	**Combination with lestaurtinib in breast cancer cells**	**Vazquez-Ortiz et al. (2014)**
**A-966492**	**BER**	**PARP1/2**	**Combination with topotecan and radiotherapy on glioblastoma spheroids**	**Koosha et al. (2017)**
**KU-55933**	**HR**	**ATM**	**Combination with radiotherapy on glioblastoma spheroids**	**Carruthers et al. (2015)**
**ETP-46464**	**HR**	**ATM/ATR, mTOR**	**Single or combination with cisplatin in platinum-sensitive and -resistant ovarian, endometrial and cervical cancer cell lines**	**Teng et al. (2015)**
**VE-821**	**HR**	**ATR**	**Combination with BETi in myc-induced lymphoma cells**	**Muralidharan et al. (2016)**
**AZ20**	**HR**	**ATR**	**Colorectal adenocarcinoma tumor cells**	**Foote et al. (2013)**
**CGK733**	**HR**	**ATM/ATR**	**Human breast cancer cells**	**Alao and Sunnerhagen (2009)**
**NU7026**	**NHEJ**	**DNA-PK**	**Combination with carbon ion irradiation in non-small cell lung cancer cell**	**Ma et al. (2015)**
**NU7441**	**NHEJ**	**DNA-PK**	**Combination with radiotherapy in non-small cell lung cancer cell**	**Sunada et al. (2016)**

Abbreviations: MGMT, O-6-methylguanine-DNA methyltransferase; APE1, AP endonuclease 1; Ref-1, redox factor-1; RAD51, DNA repair protein RAD51 homolog 1; PARP, Poly (ADP-ribose) polymerase; ATM, ataxia telangiectasia mutated; ATR, ataxia telangiectasia and Rad3-related; mTOR, mammalian target of rapamycin; DNA-PK, DNAdependent protein kinase; BETi, BET inhibitors.

Ataxia-teleangectasia mutated (ATM) and ATM-RAD3-related (ATR) protein, like DNA-PK protein, are the members of PIKK family. They work as a transducer of the DSB signal, and are involved in the repair of DNA DSBs (Weber and Ryan, 2015). A large of ATM inhibitors, including KU-55933, KU-60019, KU-59403, CP-466722, AZ31, AZ32, AZD0156, and AZD1390, have been developed and their antitumor effects have been investigated (Jin and Oh, 2019). It has been reported that human tumor cells treated with KU-55933, a specific inhibitor of the ATM kinase, could sensitize tumor cells to the cytotoxic effects of IR and DNA DSBs-inducing chemotherapeutic agents such as etoposide, doxorubicin, and camptothecin (Hickson et al., 2004; Hoey et al., 2018). KU-60019, an improved ATM kinase inhibitor, acts as a highly effective radio-sensitizer in human glioma cells (Biddlestone-Thorpe et al., 2013). AZD0156, a newly discovered ATM inhibitor, has the potential to promote the survival of leukemia-bearing mice and now is under clinical trial (Morgado-Palacin et al., 2016). Preclinical study demonstrated that ATM inhibitor AZD1390 enhanced the radiosensitivity of tumor cells and extended animal survival in preclinical brain tumor models (Durant et al., 2018). AZD1390, as a radiosensitizer, is now undergoing two clinical trials in patients with brain cancer (NCT03423628) or non small cell lung cancer (NCT04550104). Many inhibitors aiming at both ATM and DNA-PK have been reported to have great potential as a chemo- and radiotherapy sensitizing agents in cancer therapy (Powell and Bindra, 2009).

The cell cycle checkpoint kinases CHK1 and CHK2 are downstream substrates of ATM /ATR, which act as the “central transducers” of the DDR (Pilie et al., 2019). Activation of these pathways is essential for the proper regulation of checkpoint and DNA repair (Smith et al., 2010). The ATM–Chk2 and ATR–Chk1 pathways respond to different DNA damages, ATM is activated at DSBs, whereas ATR is recruited to tracts of ssDNA (Di Benedetto et al., 2017). Subsequently, CHK1 and CHK2 activated by ATR and ATM respectively upon their recruitment to DNA damage sites. Protein kinase WEE1 functioned as furthest downstream in ATR/CHK1 pathway, which is indirectly regulated by DNA damage (Cleary et al., 2020). WEE1 actives the G2/M cell cycle checkpoint by impeding cyclin-dependent kinase 1 and 2 (CDK1/2) activity, thereby inducing cell cycle arrest and promoting DNA damage repair. Inhibition of WEE1 causes aberrant DNA replication and replication-dependent DNA damage in cells by suppressing CDK2 (Guertin et al., 2013). Recently, compounds targeting CHK1 are currently in clinical trials (Table 1). The first-in-class WEE1 kinase inhibitor AZD1775 is also undergoing a series of clinical trials as monotherapy or in combination with other therapies (Table 1).

### mtDNA Repair Pathway

Recently, the exploration of novel anticancer strategies aiming at the differences in mitochondrial function and structure between normal cells and cancer cells has received intensive attention (Porporato et al., 2018). However, there are few studies that have discovered new anticancer approaches via targeting mtDNA repair pathway.

Like nDNA, efficient mtDNA repair pathway, especially BER pathway that mainly repairs ROS-induced lesion, may play an important role in cellular resistance to cancer therapeutic agents. MtDNA D-loop mutations were common in gastrointestinal cancer and correlated with carcinoma progression (Wang B. et al., 2018). It has been found that human breast cancer cells defective of mtDNA repair are more sensitive to oxidative damage than the control cells (Shokolenko et al., 2003). Grishko V I *et al* indicated that mtDNA repair pathways played an important role in protecting cells against ROS in normal HA1 Chinese hamster fibroblasts (Grishko et al., 2005). Another study clarified that mtDNA repair capacity was important for cellular resistance to oxidative damage by increasing their viability following exposure to oxidative stress (Shokolenko et al., 2003). Ueta E *et al* demonstrated that downregulation of the mtDNA repair-associated molecules, mitochondrial transcription factor A (mtTFA) and Polγ by using inhibitors of PI3K/Akt signaling in oral squamous cell carcinoma cells (OSC) increased the susceptibility of radio-sensitive OSC cells and radio-resistant OSC cells to gamma-rays (Ueta et al., 2008). This observation implied that PI3K/Akt signal inhibitors can suppress mtDNA repair capacity. Thus, these inhibitors combined with ionizing irradiation or chemotherapeutic drugs may be utilized as an effective strategy in cancer therapy.

DNA glycosylases are involved in the initiation step of BER that recognizes and removes the abnormal base (Anderson and Friedberg, 1980). 8-OxoG-recognizing DNA glycosylase 1 (OGG1) is an important DNA glycosylase for repair of 8-oxoguanine (8-oxoG), which is one of the major DNA lesions both of the nDNA and mtDNA, especially in mtDNA (Rachek et al., 2002). It has been found that tumor cells harboring overexpressed recombinant OGG1 were more proficient at repairing of oxidative damage to mtDNA, and had increased cellular survival under oxidative stress (Rachek et al., 2002; Yuzefovych et al., 2016). We previously found that Sirt3, a major mitochondrial NAD^+^-dependent deacetylase, physically associated with OGG1 and deacetylated this DNA glycosylase, and that deacetylation by Sirt3 prevented the degradation of the OGG1 protein and controlled its incision activity (Cheng et al., 2013). We further showed that regulation of the acetylation and turnover of OGG1 by Sirt3 played a critical role in repairing mitochondrial DNA (mtDNA) damage, protecting mitochondrial integrity, and preventing apoptotic cell death under oxidative stress. We observed that following ionizing radiation, human tumor cells with silencing of Sirt3 expression exhibited oxidative damage of mtDNA, as measured by the accumulation of 8-oxoG and 4,977 common deletion, showed more severe mitochondrial dysfunction, and underwent greater apoptosis, in comparison to the cells without silencing of Sirt3 expression. Our results not only reveal a new function and mechanism for Sirt3 in defending the mitochondrial genome against oxidative damage and in protecting from the genotoxic stress-induced apoptotic cell death, but also provide evidence supporting a new mtDNA repair pathway. Recently, researchers also proved that overexpression of mitochondrial OGG1 decreased breast cancer progression and metastasis (Yuzefovych et al., 2016).

In conclusion, combination of DNA repair pathway inhibitors with anticancer agents may enhance the tumor sensitivity to certain chemotherapeutic drugs and radiation. More effective and less toxic DNA-damaging agents have been developed and carried out in preclinical studies (Table 2). Based on the preclinical data, a number of clinical trials have been launched to test whether targeting DNA repair pathways can reinforce the efficacy of some anticancer drugs and benefit cancer patients (Table 1).

## The Relationship Between DNA Repair Pathways and Cancer Therapeutic Resistance

Resistance to cancer therapy remains the leading cause of treatment failure in cancer patients. DNA repair capacity (DRC) of tumor cells has been known to involve in drug resistance, including chemoradiotherapy, targeted therapy and immunotherapy. DNA damage inducing drug cisplatin is one of the most widely employed chemotherapeutic drugs. In a murine model of human lung cancer, tumor cells were initially effective with cisplatin treatment, but resistant emerged after prolonged treatment (Oliver et al., 2010). Cisplatin-resistant tumor cells exhibited higher level of DNA damage repair related genes and DRC, inhibition of NER pathway significantly enhanced the sensitivity of tumor cells to cisplatin (Oliver, Mercer, 2010; Wang et al., 2011). Low expression of 53BP1, a DDR protein involved in NHEJ, was associated with higher local recurrence in triple negative breast cancers (TNBC) patients treated with breast-conserving surgery and radiotherapy, indicating that 53BP1 may be a predictor of radio-resistance (Neboori et al., 2012). PTEN Y240 phosphorylation induced by ionizing radiation (IR), a standard treatment for glioblastoma (GBM) patients, promoted therapeutic resistance by enhancing DNA repair (Ma et al., 2019). Inhibiting DNA repair kinases could also prevent doxorubicin (DOX) resistance in breast cancer cells (Stefanski et al., 2019). Abnormal DNA repair activity was found in CDK4/6 inhibitors palbociclib-resistant breast cancer cells, whereas PARP inhibitors, olaparib and niraparib treatment could significantly inhibit palbociclib-resistant cancer cell viability (Kettner et al., 2019). In the recent years, immunotherapy is a major breakthrough in the field of cancer treatment. Therefore, the role of DDR in tumor immunotherapy has attracted much attention. Studies have shown deficiency of a specific DNA repair pathway was associated with immune checkpoint blockade (ICB) response. For example, MMR has been reported as a critical biomarker of response to immune checkpoint inhibitors in cancer (Le et al., 2017). Alterations in genes encoding MMR proteins often contribute to frameshift mutations, resulting in neoantigen generation (Germano et al., 2017). Phase II clinical trials proved that mismatch repair–deficient tumors exhibited higher responsive to PD-1 blockade compared with mismatch repair–proficient tumors(Asaoka et al., 2015). Based on lines of pre-clinical and clinical evidence, the US Food and drug Administration (FDA) has approved anti-PD-1 antibodies for the treatment of patients with MMR-deficient (Ruiz-Bañobre and Goel, 2019). On the contrary, researchers also found that colorectal cancer (CRC) patient with DNA mismatch repair deficiency (dMMR)/a high-level of microsatellite instability (MSI-H) exhibited intrinsic resistance to immune checkpoint immune checkpoint inhibitor (Gurjao et al., 2019). Metastatic urothelial carcinoma (mUC) shown relatively low response rates to PD-1/PD-L1 blockade (15–24%), whereas the presence of DDR gene mutations is a potential marker of clinical benefit from anti-PD-1/PD-L1 immune checkpoint inhibitors in mUC (Teo et al., 2018). Preclinical studies have also revealed that suppression of PARP induced PD-L1 expression and consequently caused immunosuppression (Jiao et al., 2017). Researches also elucidated that PARP inhibitor olaparib enhanced CD8^+^ T-cell recruitment and activation by activating the cGAS/STING pathway in BRCA1-deficient triple-negative breast cancer (Pantelidou et al., 2019). Therefore, multiple combination studies involving immune checkpoint inhibitors with DDR inhibitors are undergoing clinical trials, such as combination PARP inhibitor Niraparib and anti-PD-1 antibody pembrolizumab in patients with triple-negative breast cancer or ovarian cancer (NCT02657889). In the phase I, multi-center, dose-escalation study, patients with advanced solid tumors will receive WEE1 inhibitor AZD1775 (Adavosertib) in combination with MEDI4736 (durvalumab) (NCT02546661). These studies suggest that DRC plays a key role in cancer therapy resistance, therefore, evaluation of DNA repair phenotype before treatment could be of great value in clinical management of clinical therapeutic drugs or modalities.

A number of DDR inhibitors have currently come to market or under clinical development. PARP inhibitors are the first clinically approved DDR drugs based on the concept of “synthetic lethal” (Lord and Ashworth, 2017). PARP inhibitors have been widely used for cancer patients with BRCA1/2 mutation or HRR deficiency and showed promising clinical activity. However, resistance inevitably developed in the majority of patients and led to treatment failure. The mechanism of resistance to PARP inhibitors can be innate or acquired though clinical and preclinical studies. Preclinical studies demonstrated that overexpression of P-glycoprotein drug efflux transporter implicated in intrinsic resistance to Olaparib (Henneman et al., 2015). Resumption of PARformation due to poly (ADP-ribose) glycohydrolase (PARG) depletion conferred acquired resistance to PARP inhibition in BRCA2-deficient tumor cells (Gogola et al., 2018). PARP1 p. T910A mutation could override PARP1 inhibition promoted the secondary failure of Olaparib treatment (Gröschel et al., 2019). Another mechanism leading to resistance may restoration of HRR function or re-construction of replication fork stability by increasing RAD51 expression or re-expressing BRCA1/2 (Ter Brugge et al., 2016; Quigley et al., 2017; Clements et al., 2018; Lim et al., 2018; Marzio et al., 2019). Upregulation of certain oncogenic pathways such as Wnt/β-catenin signaling pathway or DDR related protein may also confer cancer cells insensitive to PARP inhibitors and providing some rationale for combination strategies with PARP inhibitors (Fukumoto et al., 2019; Watson et al., 2019; Liu et al., 2020).

## Conclusion and Perspectives

Based on the relationship between DNA repair pathways and cancer development and progression, a new therapeutic strategy has emerged to increase the efficacy of DNA damaging agents through combination with inhibitors of DNA repair pathways. The inhibitors of several DNA repair pathways have been developed, and some of them are currently undergoing clinical trials. The therapeutic benefits of these agents should be further evaluated in cancer treatment, and the more specific inhibitors should be developed to reduce the adverse effect on normal tissues and cells. Many studies have demonstrated that the inhibition of DNA repair pathways may be an important way in anticancer therapies. However, we should realize that use of certain inhibitors of DNA repair pathways may have potential drawbacks. The combination of IR or chemotherapeutic agents with inhibitors of DNA repair pathway may increase the mutagenic lesions in surviving cells and lead to the development of secondary tumors. More attentions have been paid to the relationship between defective nuclear DNA repair pathway and therapeutic resistance but less about the association between the mitochondrial repair pathway and cancer cells. Due to the difference in mtDNA between cancer cells and normal cells, the development of mtDNA repair pathway inhibitors that can reduce the adverse effects to normal cells may be a more effective strategy to enhance the anticancer therapy than targeting nDNA. A better understanding on the mechanisms of mtDNA repair pathways shall facilitate the development of new effective chemo- and radiosensitizers by targeting mtDNA repair pathway in cancer therapy.
